# Serum Uric Acid and Iron Status: Exploring a Complex Interaction in Metabolic Syndrome Patients of Eastern India

**DOI:** 10.7759/cureus.70803

**Published:** 2024-10-04

**Authors:** Saumyajit Maiti, Sudipa Pal, Debabrata Chatterjee, Arkajit Dasgupta, Amrit Podder

**Affiliations:** 1 Biochemistry, North Bengal Medical College and Hospital, Siliguri, IND; 2 Biochemistry, Institute of Post Graduate Medical Education and Research, Kolkata, IND; 3 Biochemistry, Tamralipto Government Medical College and Hospital, Midnapore, IND; 4 Biochemistry, Teerthanker Mahaveer Medical College and Research Centre, Moradabad, IND; 5 Physiology, Teerthanker Mahaveer Medical College and Research Centre, Moradabad, IND

**Keywords:** ferritin, insulin resistance, iron, metabolic syndrome, uric acid

## Abstract

Background: Metabolic syndrome, a cluster of illnesses including insulin resistance, hyperlipidemia, hypertension, and central obesity, is affecting roughly a quarter of the world population. Dysregulation of iron homeostasis may be associated with insulin resistance, leading to metabolic syndrome. Uric acid is an antioxidant currently studied in relation to several metabolic disorders. It may also be interlinked with iron metabolism. Yet, data regarding the interplay between serum iron, ferritin, and uric acid in metabolic syndrome are scarce. Hence, this study aimed to identify any alteration of serum iron, ferritin, and uric acid levels in metabolic syndrome patients of Eastern India and to explore any inter-relationship between these parameters.

Methodology: A cross-sectional observational study including 103 patients suffering from metabolic syndrome and 107 age- and sex-matched healthy individuals was conducted. Subjects were evaluated for serum iron, ferritin, and uric acid levels, besides the diagnostic parameters of metabolic syndrome.

Results: Metabolic syndrome cases had higher serum iron, ferritin, and uric acid levels as compared to the controls. Serum uric acid was positively correlated with both iron and ferritin.

Conclusion: Metabolic syndrome is associated with elevated serum levels of iron, ferritin, and uric acid. Iron overload, reflected in elevated serum ferritin, can cause oxidative stress and endothelial damage, thereby predisposing to metabolic and vascular complications. Uric acid, an antioxidant, can rise in an attempt to counter oxidative stress. Metabolic syndrome patients should be periodically assessed for iron profile and uric acid to design suitable treatment protocols for better management of disease progression and alleviation of complications.

## Introduction

Metabolic syndrome constitutes a constellation of several physiological, metabolic, and biochemical derangements that predispose to several neurovascular complications such as type-2 diabetes mellitus (T2DM), atherosclerotic cardiovascular diseases, cerebral accidents, etc. [1,2]. These factors include insulin resistance, central obesity, hypertension, hypertriglyceridemia, and decreased high-density lipoprotein cholesterol [3]. Due to the drastic lifestyle changes of the modern world, such as aggressive urbanization, reduced physical activity, and greater intake of low-fiber and high-calorie foods, metabolic syndrome has rapidly emerged as a major health burden, accounting for a 1.5-fold higher risk of all-cause mortality. The worldwide prevalence of metabolic syndrome ranges from <10% to 84% depending on various socioeconomic and demographic factors. However, approximately 25% of the global population is estimated to suffer from this condition. In India, its prevalence varies from 11% to 41% at different locations [1,4]. 

Insulin is a principal regulatory hormone of glucose metabolism in the human body. Recent studies have shed light on the intricate relationship between iron metabolism and glucose homeostasis. Iron overload has been associated with the development of metabolic syndrome. Despite the pathophysiology being unclear, higher expression of iron transport proteins in pancreatic beta cells, oxidative stress-induced endothelial damage, beta cell apoptosis, disruption of hepatic insulin extraction leading to peripheral hyperinsulinemia, etc. have been suggested [5]. Ferritin serves a major role in regulating iron homeostasis and is an effective indicator of iron status in the body. It has been postulated that excess ferritin may itself play a role in promoting iron-induced oxidative stress and lipid peroxidation, ultimately paving the way for insulin resistance and metabolic syndrome [6].

Uric acid is produced in the human body as the end product of purine degradation. Elevated serum uric acid causes the deposition of monosodium urate crystals in joints, ultimately leading to gout. A few studies have demonstrated hyperuricemia in patients suffering from metabolic disorders such as diabetes mellitus, hypertriglyceridemia, etc. Oxidative stress forms an important basis in the pathogenesis of such diseases. Elevation of uric acid in these diseases has been suggested as a response attempting to alleviate oxidative stress due to its antioxidant properties [6,7].

However, the association of serum iron, ferritin, and uric acid with metabolic syndrome, as well as the inter-relation of these three common analytes in patients with metabolic syndrome, has not been well established. Particularly in the context of the Asian population, any study to elicit the interaction of serum uric acid with iron and ferritin in metabolic syndrome patients is scarce, and no study concerning the Indian population in this regard has been found. Under these circumstances, the current study aimed to estimate the serum iron, ferritin, and uric acid status among Eastern Indian patients with metabolic syndrome compared to their healthy counterparts and to explore if there was any inter-relationship between these parameters among these patients.

## Materials and methods

Study design

This cross-sectional observational study follows the STROBE guidelines [8] and was conducted on obtaining the prerequisite ethical approval from the Institutional Ethics Committee, Institute of Post Graduate Medical Education and Research, Kolkata, WB, IND (approval no. IPGME&R/IEC/2021/462). The collection of study subjects and relevant data, as well as the biochemical analysis, was done between October 2021 and September 2022 at the Department of Biochemistry in collaboration with the Medicine Department at the Institute of Postgraduate Medical Education and Research in Kolkata. Via total sampling, 103 patients with metabolic syndrome, as diagnosed by the International Diabetes Federation criteria [1], were selected for the study from the medicine outpatient department against 107 healthy age-and-sex-matched individuals from the employees of the hospital as well as the peers and relatives accompanying the patients. A voluntary informed consent form was signed by every participant before inclusion in the study. Pregnant women, patients having a history of acute blood loss in recent periods, or chronic debilitating diseases such as malignancy, acute or chronic infection, thyroid disorders, renal and cardiac diseases, etc., as well as patients undertaking iron, estrogen, or corticosteroid therapy, were excluded from the study. All participants were assessed for demographic (age, sex), anthropometric (height, weight, hip circumference, waist circumference, systolic blood pressure (SBP), and diastolic blood pressure (DBP)), and biochemical (hemoglobin and glycated hemoglobin (Hba1c), fasting plasma glucose, lipid profile, and uric acid, serum iron profile, and ferritin) features.

Collection of data

A collection of demographic and anthropometric data and relevant clinical history were obtained via a brief standard questionnaire [9] and physical examination. Blood pressure was measured in mm of Hg by a sphygmomanometer. Waist circumference was measured per the recommendation provided by the International Diabetes Federation, i.e., from the midpoint between the lower margin of the last palpable rib and the top of the iliac crest [10]. Following 12 hours of overnight fasting, a 10 mL venous blood sample was collected from each participant by venepuncture of the antecubital vein, maintaining strict aseptic precautions. Samples were stored in appropriate vials and, after allowing to stand for 30 minutes, were centrifuged for 10 minutes at 3500 RPM to separate the serum. However, samples collected in ethylenediamine tetraacetic acid (EDTA) vials for estimation of hemoglobin and HbA1c were not centrifuged and were immediately processed. Serum samples that were not processed within eight hours of collection were stored at -20˚C temperature for a maximum period of two weeks. Hemoglobin was measured by the fluorescent flow cytometry method via the Sysmex XN 1000 Hematology Analyzer (Sysmex Corp., Kobe, JPN), plasma HbA1c by immunoturbidimetric assay using the RX Imola autoanalyzer (Randox Laboratories Ltd., Crumlin, GBR), while serum ferritin was estimated by the chemiluminescence method via the Siemens IMMULITE 1000 Immunoassay System (Siemens Healthineers, Erlangen, DEU). All other biochemical parameters, such as fasting plasma glucose (FPG), serum cholesterol, triglyceride and high-density lipoprotein (HDL) cholesterol, uric acid, iron, and total iron binding capacity (TIBC), were estimated by the ERBA XL-600 autoanalyzer (Erba Mannheim, Mannheim, DEU). Calculation of serum low-density lipoprotein (LDL) and very-low-density lipoprotein (VLDL) cholesterol was done using Friedewald’s equation.

Statistical analysis

The data were compiled in a Microsoft Excel spreadsheet (Microsoft Corp., Redmond, WA, USA). The Shapiro-Wilk test was performed to establish that the data were normally distributed. The chi-square test and independent samples t-tests were performed to compare the groups for categorical and continuous variables, respectively. The correlation between the parameters was estimated by Pearson’s correlation coefficient (r) test. A statistically significant deviation from the null hypothesis was demonstrated by the p-value of <0.05 in each case. Microsoft Excel 2019 and SPSS Statistics version 23.0 (IBM Corp., Armonk, NY, USA) were used for statistical analysis.

## Results

The present study constituted 103 cases comprising patients suffering from metabolic syndrome and 107 normal healthy controls. The case and control groups were matched for age (p=0.081) and sex (p=0.583). The study population was analyzed for demographic factors, anthropometric measurements, and biochemical parameters. The results obtained are depicted in Table 1. Significant statistical differences between the case and control groups were found in terms of systolic blood pressure (SBP) (p<0.001), diastolic blood pressure (DBP) (p=0.023), waist circumference (p<0.001), fasting plasma glucose (p <0.001), HbA1c (p<0.001), all parameters of lipid profile (p<0.001 for serum triglyceride, total, HDL, LDL, and VLDL cholesterol), serum iron (p<0.001), ferritin (p<0.001), uric acid (p<0.001), and fasting insulin level (p=0.017).

**Table 1:** Demographic, anthropometric, and biochemical comparison of cases and controls The Student's unpaired t-test was performed for statistical analysis to compare the groups. SBP: Systolic blood pressure, DBP: Diastolic blood pressure, HbA1c: Glycated hemoglobin, HDL: High-density lipoprotein, LDL: Low-density lipoprotein, VLDL: Very-low-density lipoprotein

Parameters	Controls (n=107)	Cases (n=103)	p-value
Demographic markers			
Age (years)	56.4±7.1	54.8±6.4	0.081
Gender (female/male)	56/51	50/53	0.583
Family history of metabolic syndrome	21	34	0.027
History of alcoholism, n (%)	41 (38.3)	42 (40.8)	0.716
History of smoking, n (%)	33 (30.8)	35 (34)	0.627
Anthropomorphic markers			
SBP (mm of Hg)	126.2±7.9	137.72±8.3	<0.001
DBP (mm of Hg)	83.0±5.1	89.6±5.9	0.023
Waist circumference (cm)	78.2±5.3	98.9±4.2	<0.001
Biochemical markers			
Hemoglobin (g/dl)	12.9±1.5	12.4±1.3	0.317
Serum fasting insulin (mIU/ ml)	13.6±8.4	20.1±15.8	0.017
Fasting plasma glucose (mg/dl)	106.2±14.4	134.4±20.6	<0.001
HbA1c (%)	6.0±0.23	7.1±0.42	<0.001
Serum triglycerides (mg/dl)	131.9±12.1	211.6±52.5	<0.001
Serum total cholesterol (mg/dl)	165.7±9.1	218.5±34.8	<0.001
HDL cholesterol (mg/dl)	49.4±3.6	37.8±7.2	<0.001
LDL cholesterol (mg/dl)	89.9±9.9	138.4±35.5	<0.001
VLDL cholesterol (mg/dl)	26.4±2.3	42.3±10.5	<0.001
Serum iron (μg/dl)	73.4±12.4	100.3±24.8	<0.001
Total iron binding capacity (μg/dl)	324.4±66.1	332.6±65.3	0.162
Serum ferritin (ng/ml)	47.6±14.4	84.7±26.7	<0.001
Serum uric acid (mg/dl)	4.13±0.90	5.77±0.89	<0.001

**Figure 1:**
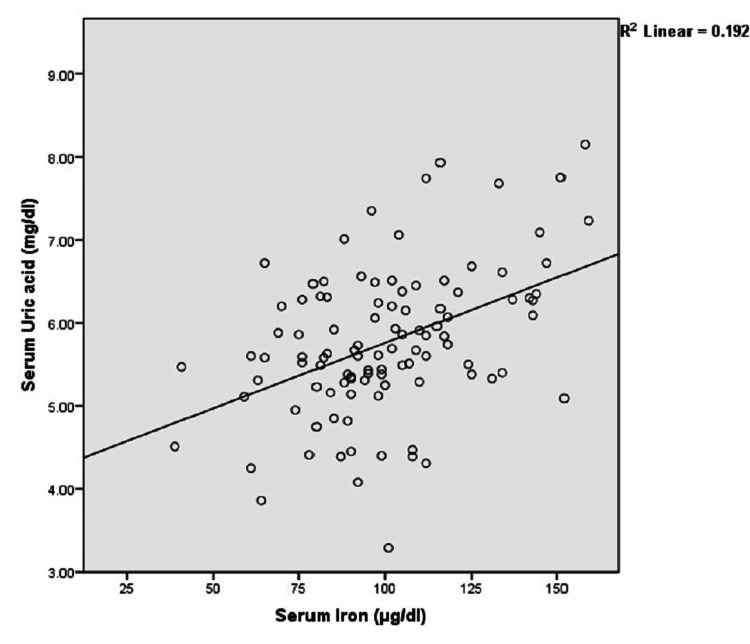
Relationship of serum iron with uric acid in cases (n=103)

**Figure 2:**
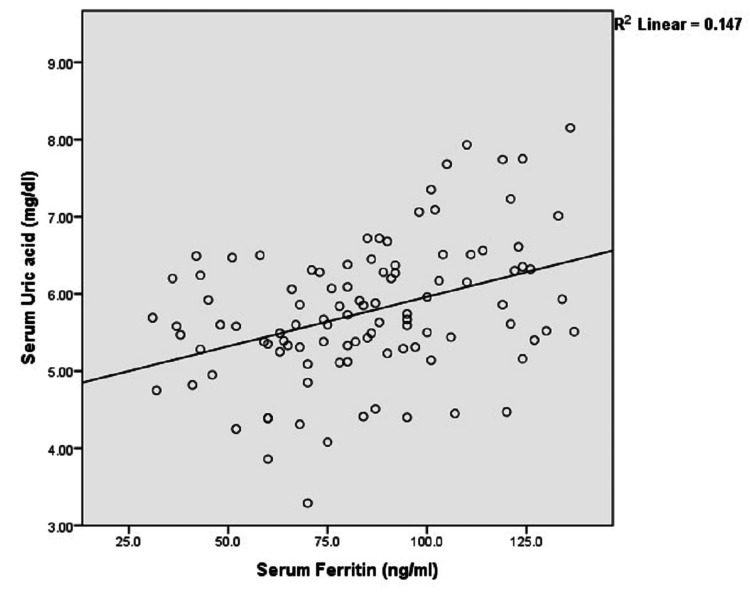
Relationship of serum ferritin with uric acid in cases (n=103)

Furthermore, male cases were compared against age-and-sex-matched healthy controls (Table 2), while comparisons between female cases and controls were also performed (Table 3), demonstrating similar results in each case. In the metabolic syndrome case group, serum iron was positively correlated with serum ferritin (r=0.399, p<0.001). Serum uric acid also demonstrated a statistically significant positive correlation with iron (r=0.439, p<0.001) as well as ferritin (r=0.384, p<0.001) as shown in Figure 1 and Figure 2, respectively.

**Table 2:** Demographic, anthropometric, and biochemical comparison of cases and controls of male gender The Student's unpaired t-test was performed for statistical analysis for comparing the groups. SBP: Systolic blood pressure, DBP: Diastolic blood pressure, HbA1c: Glycated hemoglobin, HDL: High-density lipoprotein, LDL: Low-density lipoprotein, VLDL: Very-low-density lipoprotein

Parameters	Male controls	Male cases	p-value
Age (years)	56.5±6.9	54.0±6.3	0.06
SBP (mm of Hg)	129.2±7.7	141.4±8.5	<0.001
DBP (mm of Hg)	87.3±5.7	94.5±6.3	<0.001
Waist circumference (cm)	84.3±4.3	106.2±7.1	<0.001
Serum fasting insulin (mIU/ ml)	12.9±9.1	20.7±16.1	<0.001
Fasting plasma glucose (mg/dl)	111.6±15.9	138.2±21.1	<0.001
HbA1c (%)	6.2±0.20	7.3±0.39	<0.001
Serum triglycerides (mg/dl)	131.2±12.5	204.0±54.3	<0.001
Serum total cholesterol (mg/dl)	165.9±9.7	219.2±36.8	<0.001
HDL cholesterol (mg/dl)	49.3±3.2	38.5±8.1	<0.001
LDL cholesterol (mg/dl)	90.4±10.3	139.9±36.6	<0.001
VLDL cholesterol (mg/dl)	26.2±2.5	40.8±10.9	<0.001
Serum iron (μg/dl)	74.0±13.3	100.2±26.9	<0.001
Serum ferritin (ng/ml)	45.9±14.6	85.4±24.4	<0.001
Serum uric acid (mg/dl)	4.23±0.83	5.72±0.90	<0.001

**Table 3:** Cases versus controls belonging to females The Student's unpaired t-test was performed for statistical analysis to compare the groups. SBP: Systolic blood pressure, DBP: Diastolic blood pressure, HbA1c: Glycated hemoglobin, HDL: High-density lipoprotein, LDL: Low-density lipoprotein, VLDL: Very-low-density lipoprotein

Parameters	Female controls	Female cases	p-value
Age (years)	56.3±7.4	55.5±6.6	0.569
SBP (mm of Hg)	123.5±6.9	133.8±8.8	<0.001
DBP (mm of Hg)	79.1±5.3	84.4±4.9	<0.001
Waist circumference (cm)	72.6±6.1	91.2±4.8	<0.001
Serum fasting insulin (mIU/ ml)	14.2±6.1	19.5±14.4	<0.001
Fasting plasma glucose (mg/dl)	101.3±14.8	130.4±20.1	<0.001
HbA1c (%)	5.8±0.24	6.9±0.44	<0.001
Serum triglycerides (mg/dl)	132.5±11.9	219.8±49.7	<0.001
Serum total cholesterol (mg/dl)	165.5±8.6	217.7±32.9	<0.001
HDL cholesterol (mg/dl)	49.6±3.9	37.0±6.1	<0.001
LDL cholesterol (mg/dl)	89.5±9.5	136.7±34.7	<0.001
VLDL cholesterol (mg/dl)	26.5±2.3	43.9±10.0	<0.001
Serum iron (μg/dl)	72.8±11.6	100.4±22.7	<0.001
Serum ferritin (ng/ml)	49.1±14.3	83.9±29.2	<0.001
Serum uric acid (mg/dl)	4.04±0.95	5.82±0.89	<0.001

## Discussion

As per the criteria of the International Diabetes Federation, metabolic syndrome is characterized by the presence of central obesity (waist circumference ≥90 cm in males and ≥80 cm in females of South Asian population) along with at least two of the following criteria in a person, namely elevated fasting plasma glucose (≥100 mg/dl or previously diagnosed T2DM), raised serum triglyceride level (≥150 mg/dl or specific treatment for this lipid abnormality), decreased serum HDL cholesterol (<40 mg/dl in males and <50 mg/dl in females or under treatment for such lipid anomaly) and elevated blood pressure (SBP ≥ 130 mm of Hg or DBP ≥ 85 mm of Hg or under treatment for previously diagnosed hypertension) [1].

The current study aimed to assess the serum levels of iron, ferritin, and uric acid in patients with metabolic syndrome and to explore any possible relationship among the parameters. It was found that these patients had a significantly higher mean serum iron and ferritin concentration as compared to the controls. Iron, a transitional metal, serves a critical role in the physiological functions of the human body. As a component of hemoglobin, it is chiefly involved in the mediation of oxygen transport in the blood. Iron has also been closely associated with glucose metabolism. Four of the five complexes involved in the mitochondrial electron transport chain contain iron-sulfur proteins or hemoproteins, such as cytochromes. Moreover, several mitochondrial enzymes, such as citrate synthase, aconitase, isocitrate dehydrogenase, and succinate dehydrogenase, regulating the Krebs cycle, are also dependent on the presence of iron, and their activity can be inhibited by the depletion of iron [5]. Contrarily, an excess of iron can culminate in oxidative stress, thereby facilitating mitochondrial dysfunction [11]. Production of highly reactive free radicals such as superoxide or hydroxide anions via the Fenton reaction has been attributed to the oxidative tissue damage caused by iron excess [12]. Systemic iron metabolism has also been directly interlinked with the metabolism of glucose in the body. Although the exact underlying pathophysiology remains somewhat elusive, multiple mechanisms have been proposed. Hepcidin is a negative regulator of iron metabolism. In the enterocytes, it reduces the absorption of dietary iron, while in macrophages, it traps the iron within the cells, ultimately leading to its degradation [5]. Glucose intake has been shown to augment the level of hepcidin circulating in the blood, thus reducing serum iron concentration in healthy subjects [13]. Insulin, a principal hormonal regulator of glucose metabolism, also induces the expression of hepcidin in hepatocytes, thus causing a fall in circulating iron levels [14]. Insulin is also found to promote transferrin receptor-1-mediated cellular iron uptake [15]. An excessive intracellular iron content has again been linked to insulin resistance and the development of diabetes mellitus as well as metabolic syndrome [5].

Serum ferritin is a reliable marker of body iron storage. An elevated serum ferritin level, indicating excess iron storage in the body, has been related to insulin resistance. It may be attributed to the impairment of insulin secretion due to iron deposition in pancreatic beta cells as well as a reduction in the insulin extraction capacity of the liver due to hepatic iron overload [16].

The current study also demonstrated a higher serum uric acid level in metabolic syndrome patients in comparison to the controls. Hyperuricemia has been associated with various components of metabolic syndrome, such as insulin resistance, hypertriglyceridemia, hypertension, etc. Although the underlying mechanism is not properly elucidated, it has been proposed that dyslipidemia can cause overexpression of the enzyme xanthine oxidoreductase, thus promoting the degradation of adenosine triphosphate and generating more uric acid as well as reactive oxygen species (ROS). This leads to oxidative stress, which can aggravate dyslipidemia, along with exacerbation of insulin resistance and allied metabolic changes in the body, thus triggering hyperuricemia [17]. However, the current study was unable to elicit any statistically significant correlation of uric acid with the lipid parameters. 

The current study also found that serum uric acid level was significantly correlated to serum iron as well as ferritin. The exact mechanism of such correlation is still obscure, although the antioxidant activity of uric acid in response to the oxidative stress exerted by elevated serum iron has been postulated [18]. Ferritin, courtesy of its ferroxidase activity as well as high iron-binding capacity, converts the ferrous (Fe2+) to ferric (Fe3+) ions to trap and store the majority of iron in the body. Uric acid, an important antioxidant present in human blood, can form complexes with the Fe3+ ions to inhibit the iron-catalyzed oxidative processes. Hence, a high serum uric acid may be a compensatory response to the storage of excess iron in the form of ferritin in cases of iron overload. Such an elevation in serum uric acid level in response to increased serum ferritin can be attributed to overexpression of the xanthine oxidase enzyme following iron exposure, as demonstrated by in vivo as well as in vitro studies. Iron overload and the resultant oxidative stress can also cause the secretion of pro-inflammatory cytokines such as interleukin (IL)-1, IL-6, and tumor necrosis factor-alpha (TNF-α), which may promote the enhanced expression of xanthine oxidase [18]. Furthermore, elevated serum ferritin has been linked to insulin resistance and fasting hyperinsulinemia, which are inversely related to the degree of renal excretion of urate crystals, thus contributing to a state of hyperuricemia in the presence of iron overload [19].

Limitations

There are a few limitations in the study, such as a small sample size, among others. Since serum ferritin and uric acid are both acute phase reactants and can be altered in acute inflammation, caution was exerted to minimize the potential confounder via measurement of C-reactive protein and exclusion of patients with inflammatory, infectious conditions, or liver and kidney diseases. However, other residual confounders due to unrecognized sources of inflammation could not be ruled out. Furthermore, due to the cross-sectional nature of the study, establishing a causal relationship between the condition and the analytes or the directionality of the associations could not be established.

## Conclusions

It can be concluded that metabolic syndrome is associated with elevated serum iron, ferritin, and uric acid levels. Moreover, serum iron and ferritin are positively correlated with the uric acid concentration in such patients. Iron overload and elevated serum ferritin could potentially be additional risk factors for the development of metabolic syndrome, while the potential role of uric acid in the disease needs further evaluation. More extensive longitudinal research with a larger number of samples may be warranted for establishing any causal relationship of these analytes with metabolic syndrome pathophysiology. However, in Eastern Indian patients suffering from metabolic syndrome or those at risk of developing the condition, it is of paramount importance to regularly monitor the body's iron and uric acid status besides the routine diagnostic parameters to design any necessary intervention method at the appropriate time to alleviate untoward complications.
